# New-Onset Non-convulsive Status Epilepticus in Previously Healthy COVID-19 Patient

**DOI:** 10.7759/cureus.28254

**Published:** 2022-08-22

**Authors:** Ika Noviawaty

**Affiliations:** 1 Neurology/Epilepsy, UMass Chan Medical School, Worcester, USA

**Keywords:** eeg, new-onset seizure, seizure, non-convulsive status epilepticus, covid-19

## Abstract

The novel severe acute respiratory syndrome (SARS-CoV-2) virus has spread rapidly worldwide in the last several year. COVID-19 presentation ranges widely from asymptomatic to acute respiratory failure. Interestingly, although neurological manifestations of COVID-19 have often been described in the literature, only a few cases reports describe status epilepticus associated with COVID-19 patients.

This is a case of a 52-year-old previously healthy woman who presented to the emergency department with fever, worsening cough, shortness of breath, and hypoxia. She was found to be COVID-19-positive. She developed a bilateral tonic-clonic seizure 16 days after her first symptoms appeared. Continuous video encephalogram (CEEG) showed a generalized periodic pattern with triphasic morphology. This finding is suggestive of non-convulsive status epilepticus which resolved after valproic acid loading as given. The patient is fully recovered at 6 months follow-up and seizure-free on levetiracetam 750 mg twice daily.

This case demonstrates a new onset of bilateral tonic-clonic seizure followed by non-convulsive status epilepticus associated with COVID-19 infection. As the spectrum of COVID-19 neurological manifestation has yet to be established, healthcare providers should be cognizant of the possibility of non-convulsive status epilepticus in COVID-19 patients in order to provide timely workup and management.

## Introduction

The COVID-19 pandemic caused by the novel severe acute respiratory syndrome (SARS-CoV-2) virus has spread rapidly worldwide causing unprecedented health disruption and consequences. COVID-19 presentation can range from asymptomatic, anosmia, and flu-like symptoms to acute respiratory failure, diarrhea, and heart attack. Neurological manifestations are increasingly described in the literature, however, only a few case reports describe new-onset status epilepticus associated with COVID-19 patients. Increased awareness of neurological symptoms is needed to ensure better understanding of the full spectrum of symptoms resulting from COVID-19 infection and resulting care needed to provide appropriate management.

## Case presentation

**Figure 1:**
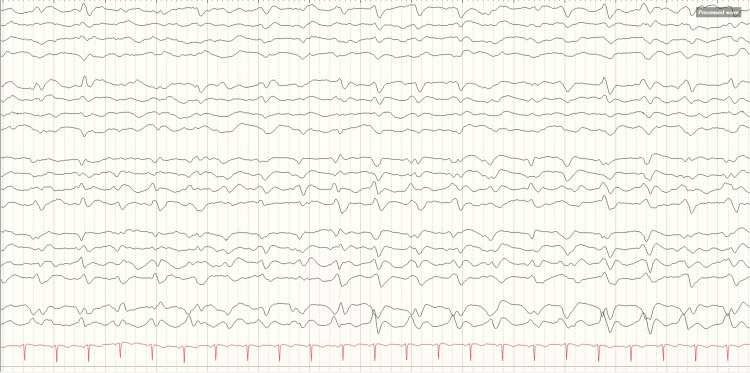
EEG showed periodic pattern

A 52-year-old previously healthy woman without history of smoking or vaping, presented to the emergency department (ED) with seven days of cough and mild shortness of breath (SOB) without evidence of hypoxia. COVID-19 testing was positive, and she was sent home for isolation. Three days after her first ED visit, she returned with worsening cough and SOB. Pulse oximetry (POx) was 80% with temperature of 39.5°C (103.1°F). She had tachypnea in the 30 breaths per minute and was unable to speak in full sentences with an unremarkable neurological examination. Chest X-ray showed bilateral infiltrates in the mid to lower lung fields. Laboratory results were significant for normal white blood cells (WBC) with lymphocytopenia 0.7 (normal 0.9-3.4 10*3/uL), arterial blood gas (ABG) pO_2_ 58 (normal 80-105 mmHg) and pCO_2_ 29.3 (normal 35-45 mmHg). POx improved to 91% with 5 L of oxygen supplementation through nasal cannula but unfortunately no improvement in pO_2_ in ABG. Tachypnea worsened which led to intubation. She was enrolled in the remdesivir trial three days after admission. Six days after intubation, the patient developed a bilateral tonic-clonic seizure that lasted for over two minutes which resolved after the administration of midazolam 4 mg intravenously (IV) and lorazepam 6 mg IV. Midazolam and lorazepam were utilized for the concern of clinical status epilepticus since bilateral tonic-clonic seizure continued past two minutes. Benzodiazepine is the first-line medication in status epilepticus treatment algorithm. Levetiracetam 1000 mg IV was given and levetiracetam 750 mg twice daily was started for anti-seizure medication maintenance while waiting for the patient to get connected to EEG. Three days of continuous video EEG (CEEG) showed evidence of waxing and waning poorly formed generalized periodic pattern (PP) in 1-2 Hz frequency (Figure 1).

**Figure 2:**
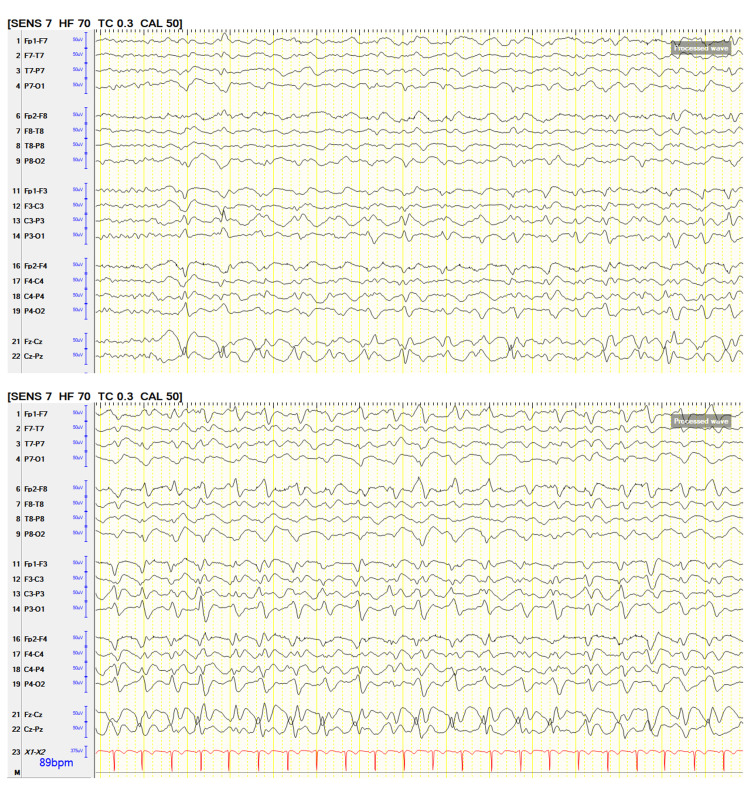
Periodic pattern showed evolution post-sedation weaning

At the time of the first seizure and CEEG initiation, the patient was on midazolam 3 mg/hour and propofol 30 mcg/kg/min. PP worsened as sedation was weaned after the first 24 hours of CEEG recording (Figure 2). Though the worsening PP frequency didn’t exceed 2.5 Hz (Salzburg criteria for status epilepticus), these EEG findings continue to be concerning since the majority of PP started showing evolution concerning ictal pattern and the patient’s altered mental status failed to improve. PP resolved after a one-time valproic acid 20 mg/kg IV was given. Clinically, the patient also gradually started waking up and following commands. MRI brain with and without contrast demonstrated multiple microhemorrhages within the corpus callosum, adjacent peri-callosal, and cerebral tissues, most pronounced in the splenium and genu segments seen in the susceptibility-weighted angiography (SWAN) sequence. The patient was discharged home after 20 days. She was seizure-free on levetiracetam 750 mg twice daily with a short-term memory deficit at 3 months follow-up which resolved at 6 months follow-up.

## Discussion

Our patient was previously healthy without seizure risk factors and developed a first-time bilateral tonic-clonic seizure in the setting of COVID-19 infection. Her seizure occurred six days after the hypoxic event that led to intubation for severe COVID-19 treatment. Clinical seizure ceased after administering additional benzodiazepine. Unfortunately, her CEEG showed PP which is could be suggestive of non-convulsive status epilepticus in the setting of persistent altered mental status. PP worsened after sedation weaning and resolved after valproic acid loading dose. Mental status examination also improved after PP was resolved. Although it is possible that a healthy individual develops seizure(s) in the setting of critical illness or hypoxia, in the setting of COVID-19 infection, we should also consider the possibility of direct viral neuronal injury (meningoencephalitis) though CSF result was negative in this patient [1-3]. Furthermore, cytokine storms in the brain as a result of viral infection can also lead to detrimental effects on the brain [2]. These factors can potentially lead to the development of new-onset seizures and status epilepticus. Furthermore, it is unclear if her seizure may have been related to a side effect of remdesivir as the trial is still ongoing and data has not been unblinded. MRI brain showed evidence of microhemorrhages in the corpus callosum which can explain the generation of the PP generalized. This is likely contributory to her memory deficit in addition to her status epilepticus and critical illness.

There is an emerging number of new-onset status epilepticus associated with COVID-19 infection. [4-10]. Healthcare providers will need to be cognizant of the possibility of non-convulsive status epilepticus in COVID-19 patients and should be considered for a complete workup for patients who have suspect symptom presentation. Timely workup and management of status epilepticus are key to improving the outcome of critically ill patients.

## Conclusions

This case study presents yet another neurological manifestation associated with COVID-19, specifically where the patient demonstrates a new onset of bilateral tonic-clonic seizure followed by non-convulsive status epilepticus associated with COVID-19 infection in a previously healthy patient. The evolution of the clinical seizure to an ictal-inter ictal continuum PP EEG morphology accompanied by persistent altered mental status captures the complexity of potential COVID-19 complications. The fact that PP worsened as sedation was weaned off and resolved after valproic acid loading dose which was then followed by neurological improvement, validated the fact that she had developed non-convulsive status epilepticus. Non-convulsive status epilepticus can often be missed in critically ill patients since they are often on sedation or have other etiology that can justify the degree of their encephalopathy. This can potentially lead to prolonged hospitalization and increased risk of morbidity or even mortality. In addition, critically ill patients also often have complicated EEG patterns such as PP which can be mistaken as encephalopathic pattern. As the spectrum of COVID-19 neurological manifestation is still being established, healthcare providers should be cognizant of the possibility of non-convulsive status epilepticus secondary to COVID-19 and be more diligent and meticulous in providing timely workup and management.
